# *Salacia reticulata* (Kothala himbutu) revisited; a missed opportunity to treat diabetes and obesity?

**DOI:** 10.1186/s12937-015-0013-4

**Published:** 2015-02-27

**Authors:** Arjuna B Medagama

**Affiliations:** Department of Medicine, Faculty of Medicine, University of Peradeniya, Peradeniya, Sri Lanka

## Abstract

**Background:**

Nearly 50% of diabetic patients worldwide use complementary medicines to treat or supplement their conventional diabetes treatment. *Salacia reticulata* (Kothala himbutu) is a woody climber used widely in the Ayurvedic system to treat diabetes and obesity.

**Objective:**

In this review I critically analyze the evidence for using *Salacia reticulata* for treating type 2 diabetes and obesity. The available evidence is described in terms of in-vitro studies, animal studies and clinical trials.

**Results and conclusions:**

In vitro studies demonstrate the ability of Salacia to inhibit intestinal alpha glucosidase. In mouse mesenteric fat it enhances the mRNA expression for hormone sensitive lipase (HSL) and adiponectin; thus increasing lipolysis and reducing insulin resistance respectively. In 3 T3-L-1 adipocytes lipogenesis factors are down regulated and lipolysis factors are up regulated with *Salacia reticulata* treatment. Animal studies and clinical trials are consistent in demonstrating improvement of glucose concentrations in the fasted and sucrose and maltose loaded states. Clinically significant reductions of HbA1C and plasma Insulin are reported with treatment of 6 weeks to 3 months. One clinical trial reported significant reduction of weight and BMI when Salacia is used in combination with vitamin D.

*Salacia reticulata* effectively improves insulin resistance, glucose metabolism and reduces obesity. A larger evidence base is required from well-planned studies to confirm its efficacy and safety.

## Background

Diabetes mellitus is a major cause of morbidity and mortality worldwide with an increasing prevalence. The WHO estimates a prevalence of 347 million people with diabetes worldwide in 2013 [1]. The prevalence is expected to double between 2005–2030 and the greater proportion of this increase would be in the low to middle income countries of Asia, Africa and South America [2].

Studies have been consistent in showing that diabetic patient adherence to current conventional treatment protocols are poor [3]. Complex treatment regimes, hypoglycaemia, patient beliefs and side effects of medications have been compelling reasons that limited patient compliance with conventional treatment [4].

On the other hand, complementary and alternative treatment systems have readily gained popularity in treating diabetes over the past few years [5]. Over 1200 plants have been claimed to be remedies for diabetes mellitus [6] and around 400 plants and compounds have been studied in detail so far. It is only recently that guidelines for investigation of such remedies have developed and few herbal remedies have been studied [7].

Similarly, body fat can have varying degrees of benefit and harm, depending on location, amount, and time of fat disposition. Among older adults, body fat is metabolically harmful, with an exception that it may offer some protection against bone loss and fractures. However, for adults, excess body fat is widely acknowledged to be associated with such diseases as type 2 diabetes, stroke, hypertension, cardiovascular disease, and arthritis [8]. In particular, abdominal fat has been associated with increased morbidity and mortality among adults [9,10].

Asia has been home for ancient healing systems and traditional healing practices that use herbal remedies [11,12] and Salacia species has been used widely for the treatment of diabetes and obesity.

Salacia genus consisting of about 120 species (e.g. *Salacia reticulata*, *Salacia oblonga*, and *Salacia prinoides*) are widely distributed in Sri Lanka, India, China, Vietnam, Indonesia and other Asian countries [12]. *Salacia reticulata* (SR), known as Kothala himbutu in Sinhalese is widely distributed in Sri Lanka and southern India. It is a large woody climber and its root and stem has been extensively used in Ayurvedic medicine for the treatment of diabetes [12]. It s also believed to contain anti-rheumatic properties and is also used for many skin related ailments in traditional healing practices [13]. *Salacia reticulata* has been studied widely for its presumed hypoglyceamic and anti-obesity effects in animal models and humans [14].

The aim of this review is to critically evaluate the evidence available from studies that used SR in vitro, animal and human studies for its hypoglycaemic and anti-obesity effects.

### Chemistry

The constituents of Salacia are numerous and varies according to the species and place of origin [12].

Multiple compounds with hypoglycaemic effects have been isolated from Salacia species. Many triterpenes, hydrocarbons and sitosterol have been isolated from roots and stem barks [13]. The root of SR has been reported to contain mangiferin (MA), kotalanol, and salacinol. These 3 compounds are primarily believed to be responsible for its hypoglycaemic effect [15,16]. In 2008 Hiromi et al. reported a thiocyclitol compound isolated from SR with hypogycaemic properties [17]. In addition, several other constituents (dulcitol, tannins, kotalagenin maytenfolic acid and soiguesterin) have been isolated from this plant, but how each constituent act on the physiological processes of the human body is not well understood [12].

### Mechanisms of action

#### Inhibition of postprandial glucose

Through bioassay guided separation of the active fraction from S. reticulata, alpha-glucosidase inhibitory effect has been identified as the primary activity responsible for its hypoglycaemic effect [13].

The intestinal ezymes, alpha-glucosidase and alpha-amylase break down starches, dextrins, maltose and sucrose into readily absorbable monosaccharides within the small intestine. Inhibition of these enzymes would cause delay in the absorption of glucose and help attenuate the postprandial glucose surges in diabetic individuals. This mechanism is currently being clinically used in alpha-glucosidase inhibitors like Acarbose [18].

Therefore extracts of SR would be expected to reduce the postprandial hyperglycaemia and hypersinsulinaemia by inhibition of poly and oligo saccharide digestion. The methanolic and/or 80% aqueous methanolic extracts from SR has been shown to inhibit the glucose levels in maltose and sucrose loaded rats. However this inhibition was not seen in glucose loaded rats, confirming the inhibitory effect of S.reticulata on the brush border enzymes [13].

Shimoda et al. demonstrated that an aqueous extract of SR dose dependently suppressed the serum glucose level induced by sucrose, maltose and alpha starch but not that induced by glucose and lactose in 1998 [19]. The same study also investigated the effect of the Salacia extract on various glucosidases and alpha-amylase prepared from rat jejunum and concluded that SR extracts strongly inhibited the action of alpha glucosidase and alpha amylase but not that of beta glucosidase.

Similarly, Yoshikawa et al. in 2002 demonstrated the ability of SR extracts to inhibit the postprandial glucose levels of sucrose and maltose loaded male Wistar rats to statistically significant levels at 0.5 and 2 hours [20]. The glucose inhibition was not seen in the glucose loaded rats. The glucose lowering effect of these studies is attributed to Kotalanol and Salacinol [13].

Hiromi et al. reported another compound isolated from SR, a thiocyclitol in 2008. They measured the sucrase and maltase inhibitory activities of the aqueous extract by using maltose and sucrose as substrates in vitro. They demonstrated that this novel compound possessed more potent alpha glucosidase inhibitory effects compared to Salacinol [17]. They also compared the postprandial glucose levels of maltose and sucrose-loaded rats following the administration of Voglibose, Salacia extract, Salacinol, and the thiocyclitol compound. The results confirmed that maltase and sucrase is inhibited by all these compounds but the inhibitory activity is several times weaker than that of Voglibose [17].

### Anti-obesity effects and Insulin resistance

#### Pancreatic lipase

Salacia species has been extensively studied for its anti-obesity effects as well. Pancreatic lipase activity is a critical enzyme for the digestion of dietary fat and hence believed to contribute towards weight reduction [14]. Another species of Salacia, (S. oblonga) has been reported to possess lipid-lowering activity. Huang et al. in 2006 demonstrated that root extract of *Salacia oblonga* inhibited olive oil induced hypertriglycerdiaemia of rats [21]. Similary hot water extracts SR also suppressed pancreatic lipase activity [22]. Thus inhibition of pancreatic lipase activity in the small intestine is believed to be the main mechanism by which postprandial hyperlipidaemia is attenuated by the Salacia root [14].

#### Differentiation of 3 T3-L1 adipocytes

Adipocytes, has long been regarded as a tissue whose primary effect is the storage of excess energy. Recent evidence suggests that it has endocrine function regulating glucose and lipid metabolism through active secretion of various adipocytokines [23]. Adipocytes are divided into premature, mature and hypertrophic adipocytes and each stage of the adipocyte secretes a different kind of adipocytokine. Hypertrophy of the adipocyte forms the basis of various metabolic diseases including insulin resistance, ischaemic cardio vascular and cerebro-vascular diseases and non-alcoholic fatty liver disease (NAFLD) [24]. Modulation of these adipocytes may therefore play a role in fat accumulation and its subsequent effects on cardiac and cerebro-vascular disease [25]. Shimada et al. in 2011 studied the differentiation of mouse-derived precursors of 3 T3-L1 adipocytes in the presence or absence of SR extracts. They demonstrated that in the presence of SR the differentiation of precursor cells into mature adipocytes was significantly inhibited. The same study demonstrated inhibitory effect on the expression of Peroxisome Proliferator Activated Receptor-gamma (PPAR), and reduction in the concentration of adiponectin. Previous studies had suggested a direct action of SR on the adipocytes in preventing obesity [26]. PPAR gamma is believed to enhance the differentiation of pre-adipocytes to mature cells. Adiponectin which is released from adipocytes improves insulin resistance and arterioscelorosis. However the changes in adiponectin levels with selacia administration still remains controversial [24,25].

#### Effect on mesenteric fat, mature adipocytes and insulin resistance

SR extract improves hypoadiponectnaemia and induces gene expression of the lipolysis enzymes, hormone sensitive lipase (HSL), adipose triglyceride lipase (ATGL) and adiponectin in the mesenteric fat. Shimada et al. in 2014 reported extracts of SR significantly increased adiponectin levels (P < 0.01) and decreased insulin levels (P < 0.05) in a dose dependent manner in a mouse model (TSOD mice) [24]. This data was supported by a concomitant increase in the mRNA expression for adiponectin in the mesenteric fat and 3 T3-L1 adipocytes compared to controls (P < 0.05). Lipid accumulation in 3 T3-L1 adipocytes was also significantly inhibited by treatment with SR. Adiponectin in turn activates AMP-activated protein kinase (AMPK), which increases the utilization of fat in liver and muscles [27]. This enhances the glucose uptake into cells and inhibition of gluconeogenesis, leading to improvement of insulin resistance [24]. Furthermore the authors conclude that activation of HSL leads to normalization of mesenteric hypertrophy and adipocytes. The study also demonstrated that SR regulates the metabolism triglycerides in mature adipocytes. This is the result of up regulation of lipolysis factors HSL, ATGL and adiponectin with SR and evidenced by the release of glycerol into the medium [24].

Akase et al. [26] showed the ability of SR extracts to suppress the individual components of the metabolic syndrome in mice. The mice receiving extracts of SR had significantly lower levels of Insulin, total cholesterol and triglycerides compared to control mice. In addition there was significantly lower levels of systolic, diastolic and mean blood pressure values in the treated mice compared to control mice. Further, histological examination of liver revealed the treated mice to have reduced fatty degeneration of the hepatocytes, reduced infiltration of inflammatory cells and single cell necrosis [26].

### Experimental evidence

#### Effects on glucose metabolism

In 1998 Shimoda et al. demonstrated that pre-treating healthy human volunteers with Salacia extract inhibited the postprandial glucose surge. They performed a sucrose tolerance test (50 g) on healthy human volunteers after pre treating them with 200 mg of an aqueous extract of the stem of SR. The study demonstrated a statistically significant suppression of the postprandial glucose values [19].

Kajimoto et al. in 2000 performed a placebo-controlled cross over trial to evaluate the effectiveness of an aqueous extract of the stem of SR on prevention and treatment of type 2 diabetes mellitus [28]. Twenty treatment naïve patients with type 2 diabetes, divided into 2 groups were given SR extract (240 mg/day) or placebo for a period of 6 weeks. The subjects were then crossed over and the experiment repeated. The results indicated significant reductions in fasting plasma glucose, HbA1C and Body Mass index (BMI) in the treatment groups.

In 2005, Jayawardena et al. performed a single centre double blind randomized placebo controlled cross over study using a patented formula of SR. Fifty one patients between the ages of 40–65 years with type 2 diabetes was selected for this study [29]. Fructosamine, HbA1C and a six point blood glucose testing were used to assess the efficacy of SR treatment. The treatment groups received the patented preparation of SR in the form of tea for a period of 3 months. The results demonstrated fall in HbA1C of 0.54 + − SD 0.93 in the treatment group compared to a fall of 0.3 + − SD 1.05 in the placebo group (P < 0.001).

Shivaprasad et al. in 2013 [30] studied the effect of SR on the serum lipids and glycaemic control in 29 patients with pre-diabetes and mild to moderate hyperlipidaemia in a double blind placebo-controlled, randomized trial [30]. Patients were divided into 3 groups to receive placebo, root or leaf extract. The treatment groups received SR at a dose of 500 mg/day for a period of 6 weeks. Fasting blood glucose (FBS) and Oral Glucose Tolerance Test (OGTT) were used to assess the response to treatment. Patients randomized to receive salacia root extract showed a significant reduction in FBS at both 3 and 6 weeks of treatment (P < 0.01). Those receiving the leaf extract showed a significant reduction in FBS (P < 0.05) only at 6 weeks. No significant differences were present in the OGTT values between groups; but a non-significant decreasing trend was observed at weeks 3 and 6.

#### Anti-obesity effects

Ofner et al. [31] studied the combined effect of SR and vitamin D on weight, body fat and the body mass index (BMI) on 40 healthy overweight or obese volunteers in an open label randomized study in 2013. The participants, aged 30–60 years were divided into 2 groups (Group A and B). Both groups received a guideline for lifestyle and fitness training for 4 weeks. The treatment group (Group B) additionally received a capsule containing SR 200 mg and 64 IU of Vitamin D3 three times a day for a period of 4 weeks. The controls (group A) received fitness and lifestyle training only. Those treated with SR and vitamin D showed 6.1% weight loss (P = 0.03) and 4.5% reduction in body fat (P = 0.01) compared to controls. A significant reduction in the BMI was also noted in group B from 31.2 to 29.3 Kg/m^2^ (P = 0.02).

A summary of the clinical trails is detailed in Tables 1 and 2.

**Table 1:** **Summary of study designs, participants and outcome measures**

	Shimoda et al. 1998 [19]	Kajimoto et al. 2000 [28]	Jayawardena et al. 2005 [29]	Shivaprasad et al. 2013 [30]	Ofner et al. 2013 [31]
Study design	Single point Sucrose tolerance test	Placebo controlled cross-over trial	Double blind randomized placebo controlled cross-over trial	Double blind randomized placebo controlled trial	Randomized open label study
Study duration	Single point intervention	6 weeks	3 months	6 weeks	4 weeks
Study population	Healthy Adults	Type 2 diabetes patients	Type 2 diabetes patients	Prediabetes (IFG & IGT)	Healthy adults
Number of participants	7	20	51	29	40
Source of SRE	Stem	Stem	Patented product	Leaves Root bark	Root powder (Exadipin)
Dose of SRE	200 mg (single dose)	240 mg/day	Not mentioned	500 mg/day	600 mg/day
Background oral hypoglycaemic therapy	None	None	Metformin Glibemclamide	None	None
End point(s)	Postprandial glucose value	FBS HbA1C BMI	HbA1C	1. FBS	
				2. Improvement in LDL-C

**Table 2:** **Summary of outcomes of clinical trials utilizing SR for diabetes and weight control**

		FBS (mg/dl) Pre intervention	Post intervention	HbA1C (%) Pre intervention	Post intervention	BMI (Kg/m2) Pre intervention	Post intervention
Kajimoto et al. (2000) [28]	Group A SRE	140 + − 14.6	125.7 + −18.6^*^	6.6 + − 0.2	6.52 + − 0.67	24.1 + − 4.4	23.5 + − 3.8^*^
	Placebo	125.7 + − 18.6 (CO)	131.6 + − 22.4^*^		6.59 + − 0.65	23.5 + − 3.8 (CO)	23.5 + − 3.8
	Group B SRE	139 + − 12.2	120 + − 16^*^	6.6 + − 0.2	6.46 + − 0.38^*^	22.8 + − 0.8	22.3 + − 2.4^*^
	Placebo		139 + −20.4		6.60 + − 0.4		22.7 + − 2.5
Jayawardena et al. (2005) [29]	SR	-	-	6.8 + −0.9	6.29 + − 1.02^*^	-	-
	Placebo	-	-	6.8 + − 0.9	6.65 + − 1.04	-	-
Shivaprasad et al. (2013) [30]	SR Root bark	115.2 + − 1.35	98.4 + −2.96^*^	-	-	-	-
	SR Leaves	115.5 + − 1.43	95.9 + − 2.5^*^ (At 6 weeks)				
	Placebo	116.1 + − 1.76	109.2 + − .5 (At 6 weeks)	-	-	-	-
Ofner et al. (2013) [31]	SR (Group B)	-	-	-	-	31.2 + − 4.7	29.3 + −4.5^*^
	Exercise only (Group A)	-	-	-	-	30.0 + − 5.3	29.4 + − 9.7

*denotes a statistical significance of P <0.05.

CO: at crossover.

#### Animal studies

Experimental studies that used animal models to demonstrate anti-hyperglycaemic and anti-obesity effects of SR are numerous.

#### Inhibition of hyperglycaemia

Serasinghe et al. [32] in 1990 demonstrated the ability of an aqueous extract of SR to inhibit hyperglycaemia using fasted, male Sprague–Dawley rats. Diabetes was induced in these rats by injection of Steptozotocin. Diabetes was confirmed by testing blood glucose and the experiments were carried out 7 days after diabetes was induced. In all cases the animals were fasted for 16 hours and the test group received SR orally as single point intervention while the control group received only water.

The results showed a significant lowering of blood glucose from 420 mg/dl (SD 49) at baseline to 294 mg/dl (SD 38) at 3 hours and 156 mg/dl (SD 59) at 4 hours in the SR group. The control diabetic rats at baseline had blood glucose of 448 mg/dl (SD 38) and following the administration of water, showed a transient reduction of blood glucose to 429 mg/dl at 3 hours followed by a rise to 496 mg/dl at 4 hours [32].

Yoshikawa et al. using male Wistar rats demonstrated the ability of a methanol extract of SR to inhibit postprandial glucose values in sucrose and maltose loaded rats. Fasted animals were administered the Salacia extract and half an hour later were given sucrose, maltose or glucose respectively and their plasma glucose was measured at 0.5, 1.0 and 2 hours later. Inhibition of plasma glucose was observed in the sucrose and maltose loaded rats but not in the glucose loaded ones [13].

The study by Kumara et al. looked at the action of different extracts of SR on the fasting plasma glucose, oral glucose tolerance and the long term diabetes control of Alloxan induced Sprague–Dawley rats [33]. When the fasted diabetic rats were acutely administered the methanol extract, there was a significant decrease in plasma glucose (P < 0.01). Similarly, there was significant improvement in glucose concentrations (P < 0.01) following oral glucose loading.

Another set of animals was fed powdered methanol fraction of SR twice daily for 120 days. They had their blood drawn for fasting plasma glucose at regular intervals, had an oral glucose tolerance test performed on day 75 of treatment and had HbA1C performed at the end of 120 days. Their values were compared against those of diabetic controls fed distilled water and normal (non-diabetic) rats.

The authors demonstrated a significant decrease of plasma glucose from day 50 through 75 to 120 (P < 0.001). The results from the oral glucose tolerance test showed a significant difference of the area under the curve of serum glucose of treated animals as compared to controls. (536 mg/dl (SD 9.91 Vs 1045 mg/dl (SD 153.07) P < 0.01). The diabetic rats that were treated with methanol extracts had an HbA1C of 5.72 (SD 0.35) compared to 9.72 (SD 0.98) in the control diabetic rats.

In 2014, Shimada et al. reported the effect of SR extract (SRE) on established obesity using TSOD mice [24]. The TSOD (Tsumura, Suzuki, Obese Diabetes) mouse, a newly developed animal model, exhibits both diabetes and obesity with marked hyperinsulinemia and hypertrophy of the pancreatic islets and might represent a common form of obese type 2 diabetes in humans. Phenotypic characterization revealed that the TSOD mouse had both insulin resistance and impaired glucose-stimulated insulin secretion. [34,35]. The study demonstrated mice fed incremental doses of SRE for 8 weeks had significant dose dependent decrease of body weight (P < 0.05). An oral glucose tolerance test performed on the TSOD mice after 8 weeks of SRE treatment revealed significant decrease of plasma glucose in the SRE fed mice compared to controls (P < 0.01). The glucose and insulin levels were significantly higher and adiponectin level was significantly lower TSOD control mice. In TSOD mice, SRE treatment significantly decreased the glucose and insulin levels and increased the adiponectin level, implying an improvement in the insulin resistance. At the same time SRE treated TSOD mice demonstrated a significant reduction (P < 0.05) of mesenteric fat.

#### Safety

Studies performed on humans failed to show any significant adverse events [28,30]. Jayawardena et al. reported dyspepsia and loose stools in patients taking S. reticulata but no severe adverse events were observed [29]. However, these patients were on regular metformin in addition to SR. As these studies did not extend beyond 3 months, the log-term safety profile remains largely unknown.

No gross pathological changes were observed from an oral single dose toxicity test of SR. Chromosomal aberration test using cultured mammalian cells failed to show any chromosomal aberrations [12].

## Discussion

The ability of S. reticulata to inhibit postprandial glucose is well documented [13,19,20,28]. These studies demonstrated the ability of SR extracts to inhibit blood glucose in sucrose and maltose loaded rats but not in glucose loaded rats, suggesting inhibition of brush border alpha glucosidase. Two potent alpha glucosidase inhibitors, salacinaol and kotalanol have been identified consistently in studies and are believed to be responsible for the attenuation of postprandial glucose in rat models as well as humans [15,16,19].

The studies by Serasinghe [32], Kumara [33] and Shimada [24] demonstrated significant reductions of plasma glucose in fasted rats. Kumara and Shimada demonstrated significant improvement of glucose values following an OGTT and in the fasted state in mice on long term treatment with SR. Serasinghe et al. and Kumara demonstrated a reduction in fasting plasma glucose in treatment naïve diabetic rats following a single administration of SR.

Generally, the mechanism responsible for reducing plasma glucose is believed to be inhibition of alpha glucosidase in the intestine by kotalanol and salacinol [13]. Although a mechanism to account for reduction of fasting glucose and glucose values following OGTT was not hitherto available, the decreasing insulin resistance (decrease of glucose and plasma insulin) in animal models on continued SR treatment demonstrated by Shimada et al. in 2014, [24] probably explains these findings. Serasinghe et al. and Kumara used a single point intervention and other mechanisms of glucose lowering needs to be considered to explain the results. They also point out that the extract was prepared in a manner similar to its Ayurvedic preparation used in diabetes for treating human subjects. Further study is warranted to replicate these findings as well as elucidate newer mechanisms of action to explain these results.

Kumara et al. also demonstrated that methanol extracts of SR near normalized the glucose values during a glucose tolerance test performed on rats receiving SR twice daily for 75 days. However as opposed to findings of Shimada, the observed basal insulin levels for the treated and control groups were not significantly different, leading them to conclude the improvement in glucose homeostasis was unlikely to be explained by the plasma insulin levels. The authors of this study postulated an extra-pancreatic mechanism for the improvement in hyperglycaemia. The study also looked at the medium term outcomes of Salacia extract, and found a significant improvement in fasting plasma glucose, HbA1C and the metabolic aberrations associated with diabetes at 120 days of treatment [33]. These studies effectively point out the ability of SR to reduce postprandial glucose, improve fasting glucose values, HbA1C and glucose handling following OGTT in animals on continued SR treatment.

However, some observations like the reduction of plasma glucose in treatment naïve fasted rats following SR administration need to be explored in detail to elucidate other mechanisms of action.

The studies performed on humans are few, but they have been consistent in demonstrating that SR reduces postprandial blood glucose and improves medium term glycaemic outcomes. The studies varied from single point interventions to studies lasting 6 weeks to 3 months. Shimoda et al. demonstrated that pre-treatment with SR inhibits glucose values in sucrose loaded healthy volunteers [19]. Two studies explored the use of SR on patients with pre-diabetes [30] and drug naïve mild type 2 diabetes patients [28] in placebo controlled trials of 6 week duration. A randomized double blind placebo controlled study explored the effectiveness of tea containing SR on glycaemic control in type 2 diabetes patients over a 3 month period using a cross over design [29]. These studies performed on normal, pre-diabetic and type 2 diabetes subjects were able to demonstrate statistically significant improvement in their respective glycaemic endpoints. Similar to the animal studies, improvement was observed in fasting plasma glucose levels as well as on measures of medium-term glucose control such as HbA1C. At present there are no studies that explored the acute effect of SR on glucose values on fasted human subjects. In the study by Kajimoto et al. the decline in HbA1C was borderline compared to the decline in fasting plasma glucose. This may be due to the short duration of the treatment arms lasting only 6 weeks, which may be inadequate to demonstrate a fall in HbA1C. Jayawardena et al. demonstrated a significant decline in HbA1C in Salacia extract treated patients at 3 months of treatment. Although inhibition of intestinal alpha glucosidase may partly explain the efficacy of Salacia in improving medium term glycaemic outcomes in humans, decreasing insulin resistance too may be responsible for the significant decline of HbA1C. Shivaprasad et al. demonstrated S. reticulata treated individuals had significant improvement of fasting plasma glucose levels, but having no significant effect on an OGTT.

The increased mRNA expression for HSL, ATGL and adiponectin leading to reduction of mesenteric fat and insulin resistance demonstrated in TSOD mice in 2014 [24] are probably key mechanisms that improve obesity and glucose handling. This probably accounts for hitherto unexplained reductions in plasma glucose levels in the fasted states and during OGTT in long-term SR treatment [32,33]. The results from the study by Shimada et al. also suggests the SR selectively improves metabolic derangements of obese individuals but not that of healthy non-obese ones.

Obese individuals have mature and hypertrophic adipocytes, leading to metabolic derangements. Hitherto, the effect of SR on these cells was not known, but the results of the Shimada study [24] is conclusive in demonstrating normalization mesenteric and adipocyte hypertrophy and increasing adiponectin secretion. At present these findings are limited to animal models and need to be explored in future human studies.

Ability of complementary medicines to have significant hypoglycaemic effect when used in isolation or when used at higher plasma glucose concentrations together with other oral agents levels has been reported [5]. This is probably attributable to their weak hypoglycaemic effects being masked by the more potent oral agents used in the treatment of diabetes. However Salacia showed significant improvement in glucose handling of both treatment naïve pre-diabetic patients as well as those on oral agents [29,30].

The contribution of postprandial glycaemia to hyperglycemia has been shown to decrease as HbA1c increased in type 2 diabetic patients. In such situations fasting plasma glucose exerts a greater effect on the HbA1C [36]. This finding may partly explain the results of Jayawardena et al. and Shivaprasad, where patients’ HbA1C and FBG were close to normal values; assuming Selacia acts primarily through inhibition of postprandial glucose.

Non-heterogeneous study population, a very limited number of subjects and the short study durations remain a common deficiency of most trials utilizing complementary medicines. In addition the methods utilized for extraction of the plant material (methanol or aqueous extraction), the particular component of the plant that’s used (E.g. stem, root, leaf) and the dose of extract used varies widely between studies. This makes interpretation of results more difficult. Indeed this remains a common problem in interpretation of trials that utilize traditional no-proprietary complementary medicines globally [5].

## Conclusions

The evidence available from animal and human studies point towards effective reduction of plasma glucose and weight in SR treated subjects. Alpha glucosidase inhibition is the most likely mechanism for the reduction of postprandial glucose. Reduction of fasting glucose, improvement in glucose handling following glucose loading and weight is most likely explained by decreased insulin resistance mediated through increasing adiponectin, suppression of lipogenesis and increased lipolysis.

Meticulously planned studies both animal and human, addressing the unresolved issues as well as studies that involve larger number of human subjects specifically addressing long-term outcomes and safety of SR treatment needs to be performed in the future.
